# Exploratory case comparison of gut microbiome functional potential in ultramarathoners differing in adiposity and finish time

**DOI:** 10.1080/15502783.2026.2725874

**Published:** 2026-09-08

**Authors:** Giulio Kai Saragiotto, Luiz Felipe Valter de Oliveira, Bárbara Geciana Tomaz dos Santos, Rafaela Nunes Sanches, Milena Merizzi de Oliveira, Fernanda Campos Freire, Rodrigo Dias de Oliveira Carvalho, Katia Sivieri, Adilson Sartoratto, Lucelia Cabral, Vasco Azevedo, Taisa Belli, Adriane Elisabete Costa Antunes

**Affiliations:** a Universidade Estadual de Campinas, Faculdade de Ciências Aplicadas (FCA/UNICAMP), Limeira, São Paulo, Brasil; b BiomeHub, Florianópolis, Santa Catarina, Brasil; c Universidade Estadual de São Paulo “Júlio de Mesquita Filho”, Faculdade de Ciências Farmacêuticas, Araraquara, São Paulo, Brasil; d Universidade Federal de Minas Gerais, Belo Horizonte, Minas Gerais, Brasil; e Universidade Estadual de Campinas, Centro Pluridisciplinar de Pesquisas Químicas, Biológicas e Agrícolas (CPQBA/UNICAMP), Paulínia, São Paulo, Brasil; f Universidade Estadual de São Paulo “Júlio de Mesquita Filho”, Departamento de Geologia Aplicada (DGA/UNESP), Rio Claro, São Paulo, Brasil; g Universidade Federal de Minas Gerais, Instituto de Ciências Biológicas, Belo Horizonte, Minas Gerais, Brasil; h Universidade Estadual de Campinas, Faculdade de Educação Física (FEF/UNICAMP), Campinas, São Paulo, Brasil

**Keywords:** Gut microbiome, ultramarathon, metabolic pathways, metagenomics

## Abstract

Understanding variations in gut microbial functional potential among endurance athletes may inform future personalized nutritional strategies. This exploratory case-comparison study aimed to investigate predicted microbial functional potential using shotgun metagenomic sequencing and fecal metabolites, in two post hoc-selected runners who represented extreme and contrasting outcomes from the same single-stage 217 km mountain ultramarathon: a normal-BMI fast-finisher and an obese slow-finisher. The main descriptive findings suggest that the normal-BMI fast finisher exhibited smaller observed differences among the analyzed enzyme-coding functions and pathways in the sample collected after race completion, with a predominance of enzyme-coding functions associated with nucleic acid-related processes and protein biosynthesis. In contrast, the obese slow-finisher showed larger observed differences in predicted functional potential between the pre- and post-race samples. Distinct fecal metabolite patterns were also observed, with selective short-chain fatty acid (SCFA) changes in the normal-BMI fast-finisher and reductions across all analyzed SCFAs in the obese slow finisher. Given the limitations of this study, including the confounding effects of adiposity, unmeasured dietary intake, and post hoc selection bias, these descriptive observations provide hypothesis-generating data rather than establishing causal relationships with performance. Taken together, these findings encourage further investigation in larger cohorts.

## 1. Background

In contemporary sports medicine, research into the modulatory role of the gut microbiome (GM) has become increasingly relevant, providing a basis for novel nutritional and clinical strategies targeting energy metabolism, post-exercise recovery, and tolerance to physiological stress. Given the established regulatory role of the GM across multiple organ systems, physical activity may modulate both its composition and metabolic output. In healthy, non-athlete individuals, these modulations are frequently described as beneficial and appear to depend, at least in part, on exercise intensity [1,2]. However, evidence suggesting that GM composition and metabolism differ across athletes with different competitive outcomes remains predominantly observational and should be interpreted cautiously, particularly when body composition and diet are not matched [3–7].

These interactions likely occur through microbial-derived metabolites that influence host physiology. Short-chain fatty acids (SCFA), For instance, have been linked to lipid and glucose metabolism in skeletal muscle [8]. Conversely, other metabolites, such as ammonia, trimethylamine (TMA), trimethylamine oxide (TMAO), and lipopolysaccharide (LPS), may adversely affect exercise performance [9]. Ultra-endurance training can alter metabolite profiles even in the absence of detectable changes in GM composition, potentially affecting athlete's performance and recovery in long-duration events [10]. Furthermore, although changes in the functional potential of the GM appear to be individualized in well-trained athletes, a general pattern has been reported across metabolic pathways related to certain amino acids, specific fatty acids, and glycolysis after ultra-endurance exercise [11]. Together, these observations suggest that examining both functional capacity (gene/pathway potential) and metabolic outputs (fecal metabolites) may be particularly informative in the context of extreme endurance stress.

Body composition is another critical determinant of ultramarathon performance and, simultaneously, a potential confounder in GM comparisons. Greater amounts of adipose tissue require greater muscular effort to run and lead to higher energy expenditure [12]. Low body fat percentage and a normal body mass index (BMI) have been associated with better ultramarathon performance [13], and lower skinfold thickness at selected anatomical sites has also been related to superior running outcomes in male athletes [12]. Therefore, marked differences in body composition may plausibly shape both performance and gut microbial ecology and should be considered when interpreting microbiome–performance contrasts.

With advances in sequencing technologies and the development of bioinformatics tools, shotgun sequencing enables more robust and reliable identification of microorganisms in a sample than amplicon sequencing, and allows for a detailed characterization of community's functional and metabolic potential [14,15]. This approach supports a more precise separation between functional potential (what the community can do inferred from genes and pathways) and metabolic products (what may be produced and detected as fecal metabolites). Despite its great potential, however, only a few studies in the literature address the application of shotgun metagenome sequencing in sports contexts. Furthermore, study provides a novel comparison of the GM metabolism of two athletes with contrasting BMI and race finish times during a single stage of a mountain ultramarathon.

Body composition is a critical independent determinant of GM ecology. Obesity independently alters GM composition and functional potential, including shifts in SCFA producers, and must be considered a major confounding factor when interpreting performance-related microbiome contrasts. For full transparency and adherence to publication ethics, it should be noted that the two athletes evaluated here were previously characterized in separate case studies [6,16]. Those studies primarily focused on characterizing the taxonomic composition of the gut microbiota. In contrast, the present investigation examines the predicted functional profile of the gut microbiome derived from shotgun metagenomic sequencing, together with fecal metabolite analyses, thereby addressing a distinct scientific question concerning the functional potential and metabolites of the gut microbiome that has not been reported previously.

Accordingly, this exploratory case-comparison study aimed to investigate the functional potential of the GM using shotgun metagenomic sequencing and to report functional shifts in fecal SCFAs and ammonia in two highly contrasting runners from the same single-stage 217 km mountain ultramarathon: a normal-BMI fast finisher and an obese slow finisher. We hypothesized that the normal-BMI fast finisher would show smaller observed differences across central predicted metabolic pathways approximately 10 min after race completion (T1) compared with baseline (T0), whereas the obese slow finisher would exhibit larger post-race variation across key pathways. We further hypothesized that these descriptive functional differences would temporally align with distinct post-race fecal SCFA and ammonia patterns. To address these aims, fecal samples were collected at three time points: T0 (one week before the race), T1 (approximately 10 min after each athlete finished the race), and T2 (one week after each athlete finished the race), and were analyzed for enzyme and pathway profiles (KEGG/EC/KO estimates) alongside fecal SCFA and ammonia concentrations. Given the inherently small sample size (*n* = 2) and the potential confounding effects of body composition and in-race intake, these findings should be interpreted strictly as hypothesis-generating.

## 2. Materials and methods

### 2.1. Study and participant characteristics

Given the exploratory, hypothesis-generating nature of this case-comparison study, it was not prospectively registered in public databases such as ClinicalTrials.gov or the Brazilian Clinical Trials Registry (ReBEC). This study was approved by the local research ethics committee (Unicamp Research Ethics Committee—number 4.179.685; July 29, 2020). Twelve athletes were pre-selected by the Brazil Ultramarathon 135 (BR135) organization and invited by email to participate. Nine athletes agreed to participate; however, only seven athletes met the inclusion criteria: male ultramarathon runners aged 44 to 70 years, training five times per week, with >5 years of ultramarathon experience and a robust race history; no antibiotic, probiotic, or synbiotic use within the last three months before the race; and no diagnosis of inflammatory bowel disease. Two athletes were excluded due to antibiotic and probiotic use, respectively.

For the present exploratory case-comparison (*n* = 2), two athletes were selected post hoc from the seven eligible participants based on extreme and contrasting race performance in the same event: the athlete who finished in the best position (7th place; ~6.45 km/h; “normal-BMI fast-finisher”) and the athlete who finished in the last position (113th place; ~2.06 km/h; “obese slow-finisher”). Table 1 summarizes their anthropometric characteristics. Notably, the performance contrast is entirely confounded by adiposity: the slow-finisher athlete was clinically obese (BMI 44.60 kg/m^2^, 40.27% body fat), while the fast-finisher athlete presented a normal-BMI (BMI 23.42 kg/m^2^, 22.55% body fat).

**Table 1.** Characterization of the athletes of the exploratory case comparation.

Anthropometric markers	Normal-BMI fast-finisher	Obese slow-finisher	Ratio
Age (years)	57	59	1.04
Height (m)	1.64	1.66	1.01
BMI (kg/m^2^)	23.42	44.60	1.90
Body fat (%)	22.55	40.27	1.79
**Skin folds**			
Triceps (mm)	5.80	34.33	5.92
Subscapular (mm)	27.00	63.33	2.35
Chest (mm)	18.00	44.83	2.49
Axillary (mm)	10.00	41.00	4.10
Abdominal (mm)	21.50	65.50	3.05
Supra iliac (mm)	8.30	46.17	5.56
Thigh (mm)	7.00	54.00	7.71
Calf (mm)	7.10	26.33	3.71
**Circumference**			
Right medial arm (cm)	28.10	42.93	1.53
Shoulder (cm)	107.5	136.67	1.27
Thorax (cm)	91.00	124.83	1.37
Waist (cm)	86.80	127.67	1.47
Abdominal (cm)	80.10	132.50	1.65
Hip (cm)	91.80	129.67	1.41
Right medial thigh (cm)	51.30	69.17	1.35
Right medial calf (cm)	35.00	46.83	1.34
**Race data**			
Finished time (h)	33:40:11	105:34:36	3.14 (difference: 71:54:25)
Average speed (km/h)	~6.45	~2.06	3.35

Note: total cumulative elevation gain for the 217-MUM was ~12,000 m.

This “extreme-case” strategy was adopted to maximize contrast regarding post-race functional GM dynamics in a real-world ultra-endurance setting. The five remaining eligible athletes (aged 44–70 years, BMI 23.13–33.09 kg/m^2^, finish times 44:35:16–92:03:18 h) were excluded. The post hoc selection of these two athletes based on extreme race finish times introduces clear selection bias, particularly given their marked differences in body composition. Furthermore, no pre-race matching was attempted for BMI, adiposity, expected performance, diet, or gastrointestinal characteristics. Consequently, the present findings are strictly descriptive of these two specific cases and cannot be generalized, even to other finishers of the same race. The post hoc nature of the design should therefore be understood as a strategy for hypothesis generation under field conditions rather than as an inferential comparison. The two athletes included in the present analysis correspond to participants previously described in separate publications [6,16]. However, the present study reports a distinct predicted functional metagenomic analysis that addresses a different research question.

### 2.2. Race setting and sampling timeline

The study was conducted during the BR135—2021 edition, a 217 km single-stage mountain ultramarathon held annually in São João da Boa Vista, SP. It is held on forest trails, asphalt roads, and gravel roads along the “Estrada Real” and the “Caminho da Fé”. With a cumulative elevation gain of ˜12,000 m combined with steep climbs and descents along the route, this race serves as a qualifying event for the Badwater Ultramarathon in the USA.

One week prior to the 217-MUM (T0), participants formalized their participation in the study by signing the informed consent form, stool samples were collected and anthropometric measurements were taken. Additional stool samples were collected approximately 10 min after each athlete finished the race (T1) and one week after each athlete finished the race (T2). It is important to note that in-race nutrition, hydration strategies, total nutrient intake, stool consistency, precise bowel transit times and gastrointestinal distress were not measured.

### 2.3. Anthropometric characterization

The anthropometric assessment was performed one week before the race. Weight was measured using a portable digital scale (G-Tech Glass 8, China) with an accuracy of ±0.1 kg. Height was measured using a portable stadiometer with an accuracy of ±0.5 cm. Eight skin folds were measured with the Lange Skinfold Caliper Adipometer with an accuracy of ±1.0 mm and pressure of 10.0 g/mm^2^ (Beta Technology, Santa Cruz, USA), and eight circumferences were measured with a tape measure with an accuracy of ±0.1 cm. All measurements were performed by the same researcher and in triplicate. Body density was estimated according to the protocol proposed by Jackson & Pollock [17], while percentage body fat was determined using the Siri equation [18].

### 2.4. Collection of stool samples

Approximately a month before the 217-MUM, athletes received kits by mail for fecal collection. These kits included sterile collection containers, a pair of disposable gloves, and a detailed manual with written and illustrated instructions for the correct procedure for collecting and storing biological material. In addition to the guidelines included in the kits, the athletes participated in personal explanations one week before the race, which were supplemented weekly by cell phone message until the day of the competition. Stool samples were collected at three time points: one week prior to the race (T0), approximately 10 min after each athlete finished the race (T1), and one week after each athlete finished the race. After the evacuation (~5 min later), the samples were collected by the athletes throughout the experimental period, with guidance from the researchers, and subsequently handed over to the researchers. After that, they were immediately stored in a high-quality styrofoam box with dry ice to preserve their integrity during transport and then (~5 h later) stored in the laboratory freezer (−20 °C). This procedure was carried out strictly in accordance with the guidelines for metagenomic sequencing and metabolite analysis. We did not record stool consistency or bowel timing.

### 2.5. Fecal short-chain fatty acids and ammonia ion analysis

A 200 mg aliquot of the fecal samples was diluted, homogenized, and centrifuged. The analysis of SCFA was performed according to the methods described by Duque and collaborators [19]. Standard curves were used to calibrate the instrument (acetic acid: 8.8 to 88 mM; propionic acid: 7.3 to 73 mM; butyric acid: 5.8 to 58 mM). To analyze fecal ammonia concentration, we followed the guidelines provided by Bianchi et al. [20]. An ion meter equipped with an ammonia-selective electrode (Hanna Instruments – HI 3221 pH/ORP/ISE Meter, SP, BRA) was used.

Replicates and summary statistics. For each fecal sample, metabolite quantification was performed in technical triplicates (i.e. repeated measurements from the same homogenized sample). Accordingly, SCFA and ammonia results are reported as mean ± standard deviation (SD) across technical triplicates. Technical replicates were used to quantify analytical variability and do not represent biological replication.

### 2.6. DNA extraction, metagenomic shotgun sequencing and bioinformatics analysis

After fecal sample collection, an aliquot of feces was transferred to a tube containing a stabilizing solution (ZSample; BiomeHub, SC, Brazil) to preserve and transport genetic material at room temperature. Total DNA was extracted using the ZymoBIOMICS DNA Miniprep Kit (Zymo Research) according to the manufacturer's instructions. Library preparation was performed with the Illumina DNA Prep Kit (Illumina Inc., USA). The final concentration of the pooled libraries was initially estimated using the PicoGreen fluorescence assay (Invitrogen, USA) and subsequently diluted for accurate quantification by real-time PCR with the Collibri Library Quantification Kit (Invitrogen, USA). Sequencing was carried out on a NextSeq 1000 platform (Illumina Inc., USA) using standard Illumina primers supplied with the kit. Paired-end (2 × 150 bp) sequencing was performed with the NextSeq 1000/2000 P1 Reagents kit (Illumina, USA), yielding an average of approximately 1 million reads per sample.

Two distinct functional profiling pipelines were used. The first contemplates quality control analysis of the raw FASTQs using the Biobakery KneadData pipeline v0.12.0 [21] (http://huttenhower.sph.harvard.edu/kneaddata), where sequencing adapter sequences and low-quality bases are removed using Trimmomatic v.0.33 [22]; decontamination is performed using the bowtie2 version 2.2 program [23], based on the human genome (NCBI accession number: GCF_000001405.26). Subsequently, Taxonomic identification is performed using MetaPhlAn 4.0.6 [24], which utilizes the ChocoPhlAnSGB version 202212 database, which uses approximately 5.1 million unique and clade-specific gene markers from approximately 1 million microorganism genomes [24]. The prediction of gene families is performed using a database from UniRef90 [25] and using the HUManN3 software [26], taking into account the taxonomic classification performed by MetaPhlAn. In this way, gene families are predicted to be stratified by taxonomy. Subsequently, these families are regrouped and counted in EggNOG database [27] and enzyme families were identified using the Enzyme Commission (EC) number. Functional data resulting from classification across various databases are analyzed based on normalized counts per million (CPM). This process is performed using BiomeHub's proprietary software.

In the second pipeline, host-derived reads were removed by mapping all quality-filtered reads against the human reference genome assembly GRCh38 using Bowtie2 [23] and then assembled de novo using MEGAHIT, a de Bruijn graph–based assembler optimized for large and complex metagenomic datasets [28]. Contigs were subsequently annotated against the Kyoto Encyclopedia of Genes and Genomes (KEGG) database to identify metabolic pathways and infer the functional potential of the microbial communities [29]. Metabolic pathway relative abundance was based on absolute gene counts found in each pathway and compared for each athlete between samples collected at T0 and T1. KEGG pathway profiles were generated by counting the number of non-redundant genes mapped to each pathway, providing a descriptive overview of the functional repertoire. The reported values should not be interpreted as normalized abundance estimates or statistically inferred differences between runners. For visualization, the metabolic pathways displayed in the bar plot were according to the difference in the number of non-redundant genes.

Data were generated in RStudio v4.5.0 (Posit PBC, MA, USA), employing the tidyverse, pheatmap, ggplot2, dplyr packages, and GraphPad Prism 9 (GraphPad Software, MA, USA).

### 2.7. Difference in normalized abundance calculation

**Figure 1.**
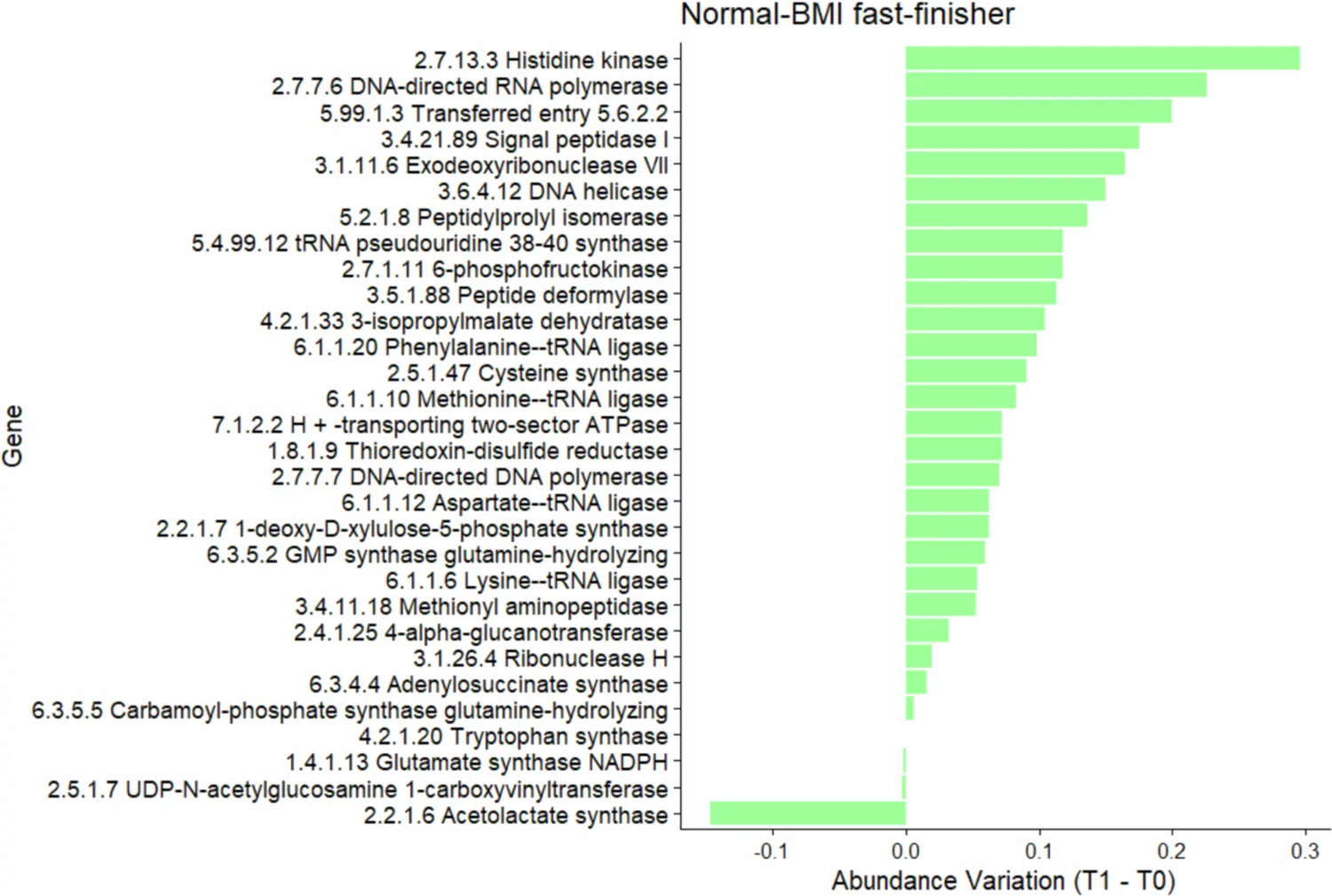
Functional enzyme profile of the normal-BMI fast finisher between post-race (T1) and baseline (T0). The bar plot represents the abundance variation in gene counts percentage. The 30 genes presenting difference in normalized abundance values are shown. Functions were annotated by enzyme (EC/KO) mapping to KEGG from shotgun metagenomic sequencing. Features are presented as descriptive rankings; no statistical testing was performed. Negative values indicate higher relative abundance at T0, while positive values indicate higher relative abundance at T1. Data are presented in CPM.

**Figure 2.**
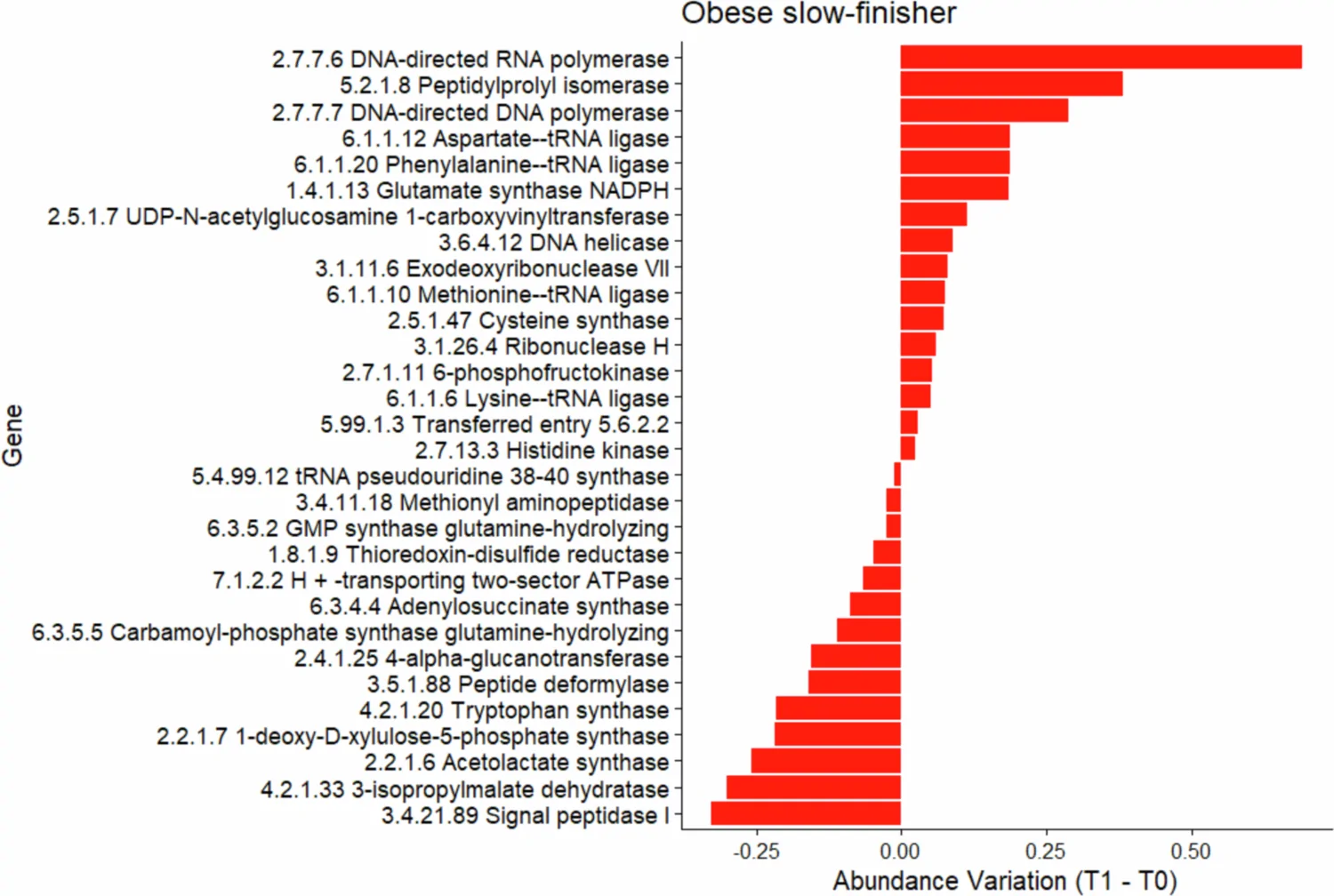
Functional enzyme profile of the obese slow-finisher between post-race (T1) and baseline (T0). The bar plot represents the abundance variation in gene counts percentage. The 30 genes presenting difference in normalized abundance values are shown. Functions were annotated by enzyme (EC/KO) mapping to KEGG from shotgun metagenomic sequencing. Features are presented as descriptive rankings; no statistical testing was performed. Negative values indicate higher relative abundance at T0, while positive values indicate higher relative abundance at T1. Data are presented in CPM.

For descriptive comparisons of functional pathways, differences in normalized abundance were calculated as the simple arithmetic difference in normalized pathway abundance (counts per million, CPM) between two time points within each athlete. Changes between post-race (T1) and pre-race (T0) samples were uniformly calculated as Δ = T1—T0. Features displayed in Figures 1,2 and 4 represent the top 30 functions exhibiting the largest absolute magnitude of change, defined as T1–T0. These values represent descriptive changes in pathway abundance and are not log-fold changes or standardized differences in normalized abundance. No inferential effect-size statistics or pathway enrichment tests were conducted; accordingly, variations are reported strictly as differences in normalized abundance or pathway aggregation.

## 3. Results

### 3.1. Overview of post-race functional shifts

Shotgun metagenomic functional profiling revealed asymmetric post-race shifts between baseline (T0) and approximately 10 min after each athlete finished the ultramarathon race (T1) in the two selected cases. Overall, the normal-BMI fast-finisher exhibited smaller observed differences among the analyzed enzyme-coding functions and KEGG pathways in the sample collected after race completion, whereas the obese slow-finisher exhibited larger observed differences across these features. In this manuscript, the term “top varying functions” refers to the features with the largest changes at T1–T0 differences in relative abundance and not to statistical significance, as no inferential testing was performed in this case-comparison design. Collectively, these patterns should be interpreted as descriptive contrasts between a normal-BMI fast-finisher and an obese slow-finisher, rather than as evidence that GM differences were linked to race performance. In Figures 1, 2, and 4, negative values indicate higher relative abundance at T1 compared with baseline, while positive values indicate higher relative abundance at T0, suggesting a reduction in these functions in the sample collected after the race.

### 3.2. Top varying enzyme-coding functions in the normal-BMI fast-finisher (T1 vs T0)

Figure 1 summarizes the top 30 enzyme-coding functions that changed in the normal-BMI fast-finisher between samples collected before and after race completion. In this comparison, the functional profile in the post-race sample showed increase in the normalized abundance of predicted gene functions involved in signaling and regulation, transcription, and repair, we observed increased abundance of enzyme-coding functions such as histidine kinase (EC 2.7.13.3), DNA-directed RNA polymerase (EC 2.7.7.6), signal peptidase I (EC 3.4.21.89), exodeoxyribonuclease VII (EC 3.1.11.6), DNA helicase (EC 3.6.4.12), 6-phosphofructokinase (EC 2.7.1.11), peptidyl-prolyl isomerase (EC 5.2.1.8), and selected aminoacyl-tRNA ligases (e.g. lysine-, phenylalanyl-, and methionine-tRNA ligases, (EC 6.1.1.6/6.1.1.20/6.1.1.10), as well as DNA cytosine-5-methyltransferase (EC 2.1.1.37). These changes were followed by a minor increase in adenylosuccinate synthase (EC 6.3.4.4) and other aminoacyl-tRNA ligases. Conversely, we observed a lower relative abundance of genes at T1 compared to T0. The main enzyme-coding function present at T0 was acetolactate synthase (EC 2.2.1.6).

### 3.3. Top varying enzyme-coding functions in the obese slow-finisher (T1 vs T0)

Figure 2 shows the variation in the relative abundance of enzyme-coding functions in the obese slow-finisher, comparing the difference between samples collected before and after race completion. Overall, we observed that this athlete exhibited a heterogeneous pattern of change in predicted microbial functional potential after the ultramarathon. The largest variations were observed in functions annotated as DNA-directed RNA polymerase (EC 2.7.7.6), peptidyl-prolyl isomerase (EC 5.2.1.8), DNA-directed DNA polymerase (EC 2.7.7.7), aspartate-tRNA ligase (EC 6.1.1.12), phenylalanine-tRNA ligase (EC 6.1.1.20), glutamate synthase NADPH (EC 1.4.1.13), UDP-N-acetylglucosamine 1-carboxyvinyltransferase (EC 2.5.1.7), and DNA helicase (EC 3.6.4.12). In contrast, some predicted functions showed negative values, indicating higher relative abundance in the pre-race sample and, therefore, lower relative abundance in the samples collected after race completion. These included signal peptidase I (EC 3.4.21.89), 3-isopropylmalate dehydratase (EC 4.2.1.33), acetolactate synthase (EC 2.2.1.6), 1-deoxy-D-xylulose-5-phosphate synthase (EC 2.2.1.7), tryptophan synthase (EC 4.2.1.20), peptide deformylase (EC 3.5.1.88), 4-alpha-glucanotransferase (EC 2.4.1.25), and carbamoyl-phosphate synthase glutamine-hydrolyzing (EC 6.3.5.5).

### 3.4. Temporal patterns across T0, T1, and T2 (heatmap)

**Figure 3.**
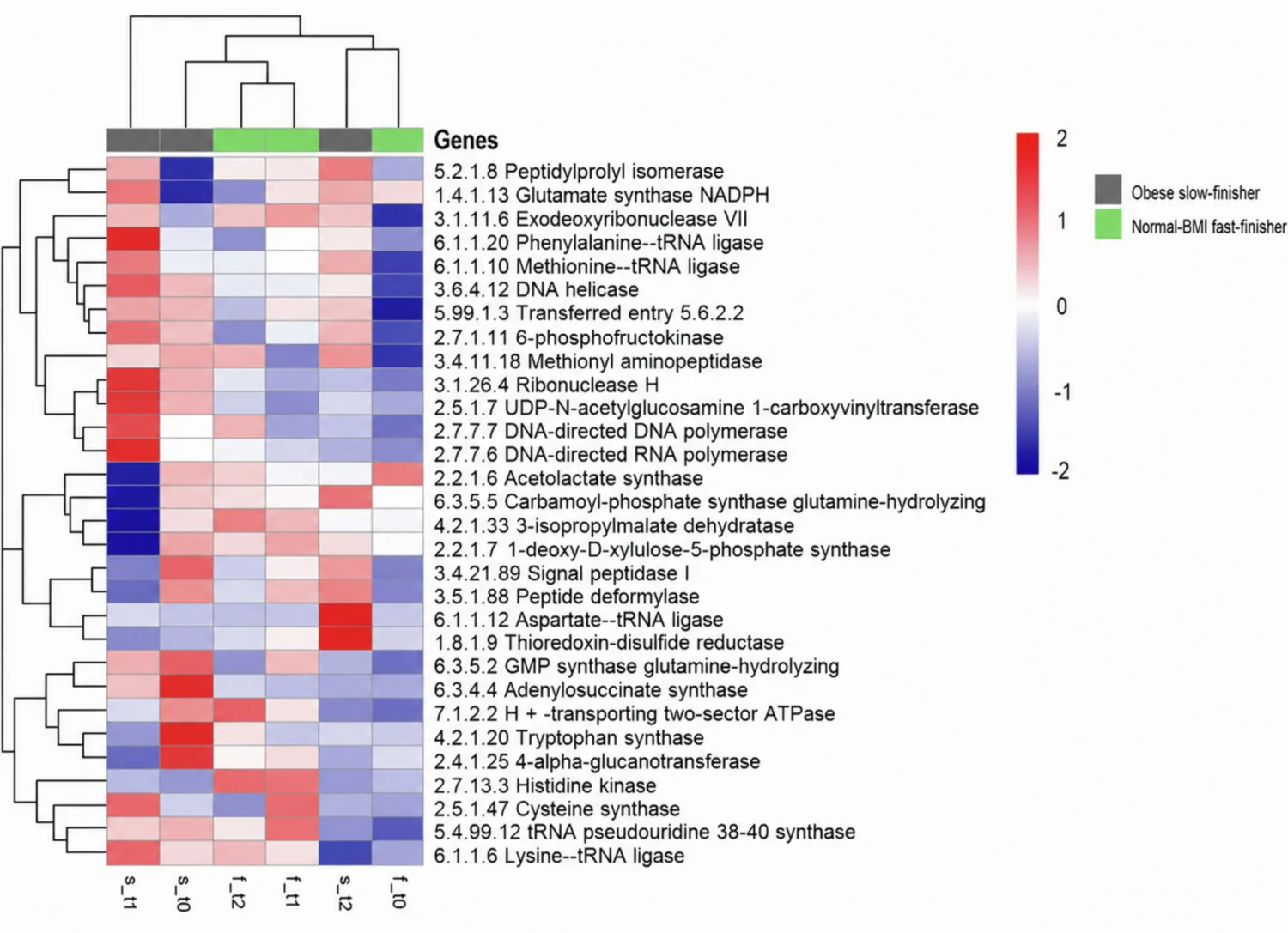
Heatmap of enzyme-coding genes with the highest abundance variation between the athletes across the different time intervals. The color scale represents the relative abundance of gene copies. Positive values (shown in red tones) indicate abundances above the mean level for a given gene across all samples, while negative values (shown in blue tones) indicate abundances below the gene mean. Values near zero (white) represent abundances close to the average relative level for that gene. Values represent row z-scores derived from log2-transformed normalized CPM abundances. Hierarchical clustering was performed using Euclidean distance with average linkage. s (obese slow-finisher) and f (normal-BMI fast-finisher); t0 (baseline), t1 (post-race), and t2 (seven days after each athlete finished the race). The features shown are descriptive and no inferential statistical testing was performed.

Figure 3 shows a heatmap summarizing the variation in selected enzyme-coding genes across athletes and time points. For clarity, the heatmap represents the relative abundance, or relative change, of gene-associated functions rather than “expression,” since the data are derived from shotgun metagenomic functional profiling. In the normal-BMI fast-finisher, several predicted functional potentials involved in nucleic acid processes and protein biosynthesis showed increased relative abundance in the sample collected at T1 and/or T2 compared with T0, whereas some carbohydrate metabolism-related functions showed moderate reductions by T2. Conversely, the obese slow-finisher exhibited wider oscillations between samples collected before and after race completion across multiple predicted functional potential genes, including replication/transcription-related functions, carbohydrate metabolism, amino acid-related pathways, and redox-associated enzymes. Taken together, these descriptive patterns indicate that the normal-BMI fast-finisher exhibited smaller observed differences among the analyzed predicted functional potential features in the sample collected after race completion, whereas the obese slow-finisher exhibited larger observed differences. However, these findings should be interpreted as exploratory and hypothesis-generating rather than evidence of altered gene expression or metabolic activity.

### 3.5. Pathway-level predicted functional potential profiles (T1 vs T0)

**Figure 4.**
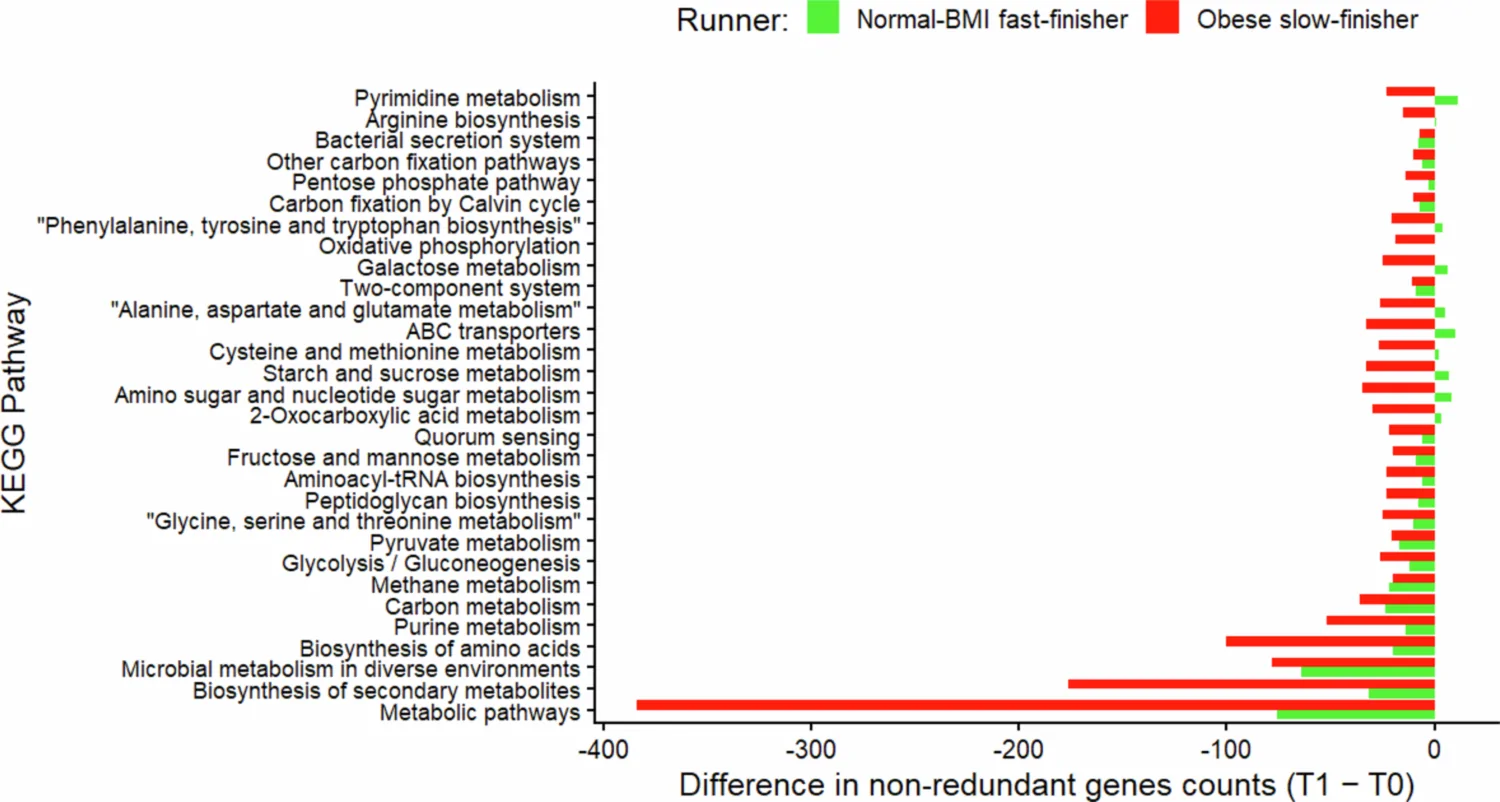
Descriptive functional profile of metabolic pathways for the normal-BMI fast-finisher and obese slow-finisher at time points T0 and T1. The bar plot shows the difference in absolute gene counts within each functional category for each athlete between T0 and T1. The metabolic pathways displayed in the bar plot were selected according to the difference in normalized abundance values and are shown as descriptive features only; no statistical inferential testing was performed. Negative values indicate higher pathway-associated gene counts at T0 than at T1, whereas positive values indicate higher pathway-associated gene counts at T1 than at T0. Data are presented in CPM.

At the pathway level (Figure 4), functional profiling based on KEGG pathway aggregation revealed marked differences between pre-race and post-race samples in both athletes. Overall, the obese slow-finisher exhibited greater post-race variation in normalized pathway abundances across several predicted metabolic functions. The most pronounced differences occurred within broad functional categories, including general metabolic pathways, biosynthesis of secondary metabolites, microbial metabolism in diverse environments, and amino acid biosynthesis. This pattern was similarly evident in pathways essential to energy metabolism, such as carbon metabolism, pyruvate metabolism, glycolysis/gluconeogenesis, pentose phosphate pathway, and oxidative phosphorylation. Furthermore, the obese slow-finisher displayed larger alteration in predicted pathways associated with amino acid and nitrogen metabolism, including glycine, serine, and threonine metabolism; cysteine and methionine metabolism; alanine, aspartate, and glutamate metabolism; arginine biosynthesis; and phenylalanine, tyrosine, and tryptophan biosynthesis. In contrast, the normal-BMI fast-finisher showed smaller observed differences across most analyzed KEGG pathways between samples collected before and after race completion.

### 3.6. Fecal short-chain fatty acids and ammonia ions

**Figure 5.**
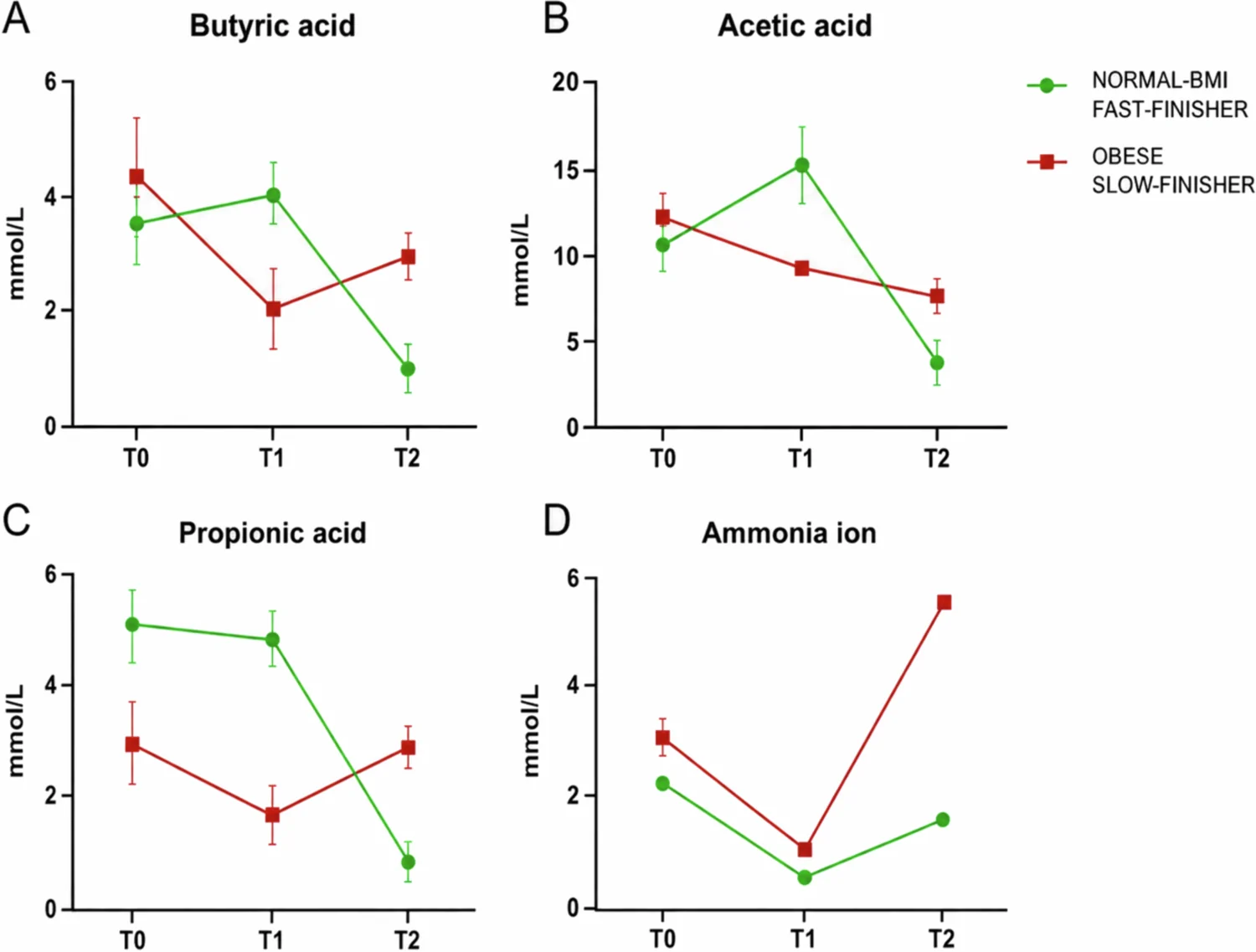
The concentrations of all investigated fecal short-chain fatty acids in the normal-BMI fast-finisher and obese slow-finisher. (A) Concentration of butyric acid in both athletes. (B) The concentration of acetic acid in both athletes. (C) The concentration of propionic acid in both athletes. (D) The concentration of fecal ammonia ions in both athletes. T0: seven days before the race; T1: approximately 10 min after each athlete finished the race; T2: seven days after each athlete finished the race. All data are expressed as the mean and standard deviation of technical triplicates. The features shown are descriptive and no inferential statistical testing was performed.

Figure 5 shows that the normal-BMI fast-finisher exhibited higher fecal concentrations of all measured SCFA (acetate, propionate, and butyrate)—than the slower runner in the post-race sample. In the faster runner, acetate concentration increased slightly in the post-race sample compared to the sample collected before the race, followed by a notably decrease one week after the race finished. Propionate concentration decreased at T1 and showed a pronounced reduction in the 7 days post-race sample compared to the baseline sample. Butyrate concentration showed a moderate increase in the T1 sample and markedly decreases in the 7 days post-race sample compared with baseline sample. Fecal ammonia ions concentration also decreased in this athlete, with a moderate reduction between samples collected after and before race completion.

In the obese slow-finisher, all measured SCFA decreased in the sample collected after the race ended. Acetate concentration showed a moderate decrease at T1 and remained relatively stable at T2. We observed a moderate decrease in the propionate concentration in the post-race sample, and returned close to baseline in the sample collected 7 days after race completion. Butyrate concentration showed a more pronounced decrease in the post-race sample, followed by a moderate increase in the T2 sample compared with the post-race sample. Fecal ammonia ions concentrations were lower in the normal-BMI fast-finisher across all time points. Although both runners showed a reduction in fecal ammonia ions in the post-race sample, the obese slow-finisher exhibited a marked increase in the 7 days post-race sample compared to the baseline sample.

## 4. Discussion

Importantly, the biological specimens analyzed in the present investigation were derived from the same two runners evaluated in our group's prior publications [6,16]. However, whereas our previous studies focused exclusively on gut microbial taxonomic composition, the current work expands upon those foundational findings by integrating functional metagenomic capacity with fecal metabolite analysis. Consequently, this study serves as a functional complement to our prior compositional characterizations of individual subjects.

In well-trained athletes, changes in the predicted functional potential of the GM are expected to be highly individualized, although recurrent patterns may occur in predicted pathways related to amino acid, fatty acid, and carbohydrate metabolism after ultra-endurance exercise [11]. In this descriptive comparison, the normal-BMI fast-finisher exhibited smaller observed differences among the displayed pathways in the sample collected after race completion, whereas the obese slow-finisher showed larger observed differences across KEGG pathways. These predicted functional contrasts co-occurred with distinct fecal metabolite patterns, including case-specific variation in SCFA in the post-race sample, with selective changes in the normal-BMI fast-finisher and reductions across all measured SCFA in the obese slow-finisher. However, these observations should not be interpreted as evidence that GM functional differences contributed to race performance. Rather, they should be understood as descriptive patterns observed in two post hoc-selected cases that were simultaneously contrasting in finish time and body composition.

Several changes were observed in the post-race sample, including predicted and annotated gene families related to signaling, transcription, protein processing, translation-related functions, and genome-related mechanisms. In the normal-BMI fast-finisher, the relative abundance of histidine kinase (EC 2.7.13.3) was higher at T1 than at baseline, suggesting increased predicted functional potential related to microbial environmental sensing. DNA-directed RNA polymerase (EC 2.7.7.6) also showed an increase in normalized abundance in the sample collected after race completion in both athletes, with a larger observed difference in the obese slow-finisher. However, because shotgun metagenomics does not measure gene expression, this finding should be interpreted only as a higher relative abundance of gene families encoding RNA polymerase-associated functions, rather than as evidence of increased transcriptional activity [30]. In the normal-BMI fast-finisher, higher relative abundance in the post-race sample was also observed for enzyme-coding functions such as signal peptidase I, peptidyl-prolyl isomerase, DNA cytosine-5-methyltransferase, exodeoxyribonuclease VII, DNA helicase, tRNA pseudouridine synthase, and selected aminoacyl-tRNA ligases suggesting variation in predicted functional potential related to protein processing, translation, nucleotide-related processes, and genome-associated mechanisms. A previous metagenomic study suggests that physical exercise and fitness level may be associated with differences in microbial functional pathways [11], but these findings should be interpreted with caution. However, BMI and body fat percentage are other factors that may have influenced these findings more than the level of training.

In both athletes, gene families assigned to carbohydrate and amino acid metabolism also showed athlete-specific patterns. In the normal-BMI fast-finisher, selected functions related to carbohydrate metabolism and translation-associated processes showed higher relative abundance in the sample collected after race completion, including 6-phosphofructokinase, peptide deformylase, and several aminoacyl-tRNA ligases. In contrast, the obese slow-finisher showed higher relative abundance in the post-race sample for some genome- and translation-associated functions, such as DNA-directed RNA polymerase, DNA-directed DNA polymerase, peptidyl-prolyl isomerase, aspartate-tRNA ligase, and phenylalanine-tRNA ligase, while several other functions related to protein processing, amino acid biosynthesis, and carbohydrate metabolism showed lower relative abundance after the race finished [31–33]. Therefore, these findings suggest distinct post-race patterns in predicted microbial functional potential between the two athletes. At the pathway level, the obese slow-finisher showed a broader reduction in the relative abundance of several KEGG pathways in the sample collected after the race ended, including metabolic pathways, biosynthesis of secondary metabolites, microbial metabolism in diverse environments, biosynthesis of amino acids, glycolysis/gluconeogenesis, pyruvate metabolism, pentose phosphate pathway, oxidative phosphorylation, and pyrimidine metabolism. In contrast, the normal-BMI fast-finisher displayed smaller observed differences among the analyzed KEGG pathways between samples collected before and after race completion. This pattern reflects smaller magnitude shifts within the predicted functional repertoire rather than evidence of gut microbiota-mediated energetic or anabolic support, and may be substantially confounded by baseline differences in BMI.

The fecal metabolite data indicated that the normal-BMI fast-finisher had higher SCFA concentrations in the post-race sample than the obese runner, whereas the obese slow-finisher exhibited reductions in all quantified SCFA in the sample collected after the race ended. In this exploratory case comparison, this pattern co-occurred with the larger observed differences across pathways in the obese slow-finisher. However, these findings are strongly limited by unmeasured factors such as BMI-related GM ecology, race duration, pre-race and in-race dietary intake, carbohydrate and fiber exposure, supplement use, last-meal timing, hydration status, gastrointestinal distress, and differences in gastrointestinal transit. The two athletes differed by more than 70 h in finish time; therefore, T1 sampling occurred after markedly different cumulative physiological and nutritional exposures. Higher fecal SCFA concentrations should not be interpreted as evidence of better performance or as a mechanistic explanation for the faster race outcome. Fecal SCFA represent the net balance among microbial production, host absorption, intestinal transit time, stool water content, luminal pH, diet, hydration status, and sampling time. Therefore, the correspondence between metagenomic profiles and fecal SCFA observed here should be interpreted as convergent descriptive evidence requiring targeted validation.

Previous studies provide biological context for these observations. Endurance exercise training has been reported to increase SCFA concentrations in lean individuals, but not in obese individuals, potentially due to differences in SCFA-producing bacteria [1]. Athletes may also exhibit higher fecal SCFA concentrations, particularly acetate, possibly related to higher dietary fiber intake and higher levels of SCFA-producing bacteria [3,9,11,33]. Scheiman et al. [4] showed that *Veillonella* species can metabolize exercise-derived lactate and generate SCFA in experimental models; however, such mechanisms should not be directly extrapolated to the present study, which did not measure lactate cross-feeding, microbial gene expression, systemic acetate availability, or host performance mechanisms. Importantly, obesity itself is associated with reduced diversity, altered energy-harvest pathways, and reduced abundance of several butyrate-producing taxa [34,35]. Therefore, the broader pathway contractions and lower SCFA levels observed in the obese slow-finisher may plausibly reflect obesity-related GM ecology, prolonged race exposure, or both, rather than race performance per se [36,37].

In previous studies from our group, in sample collected after completion of the BR135, we observed an increase in Veillonellaceae and other SCFA-producing taxa in the normal-BMI fast-finisher, alongside reductions in this taxon in the obese slow-finisher [6,16]. An independent study involving ultramarathon runners with normal-BMI did not report consistent changes in *Veillonella* abundance, but observed reductions in selected SCFA-producing species among lower-performing athletes after a ~100-km race [38]. Together, these findings suggest that SCFA-related taxa and pathways may vary across athletes characteristics and race contexts. Nevertheless, whether these patterns reflect performance-related mechanisms, dietary intake, body composition, race duration, or other physiological and behavioral factors remains uncertain. Finally, patterns related to ammonia should also be interpreted with caution. Although ammonia, TMAO, and LPS have been discussed as potentially having negative impacts on performance, elevated concentrations of these ions appear to have links between BMI, diet, gut microbial metabolism, inflammation and fatigue, the present data do not allow causal interpretation. Ammonia concentrations may have been influenced by dietary protein intake, carbohydrate availability, hydration, gastrointestinal transit, renal processing, race duration, or sampling time. Therefore, both SCFA and ammonia findings should be interpreted as descriptive fecal metabolite observations, rather than as evidence of performance-related microbial mechanisms [39–42]. Overall, this exploratory case-comparison study suggests that two athletes with highly contrasting outcomes in a 217-km ultramarathon exhibited distinct predicted microbial functional potential and fecal metabolite profiles between samples collected before and after the race.

### 4.1. Limitations

This exploratory case-comparison presents substantial limitations that restrict inferential interpretation. Additionally, the event was conducted under strict COVID-19-related restrictions, which directly affected the experimental design and field logistics, including the inability to collect samples at the start line, staggered individual starts, and participant withdrawal. First, the post-hoc extreme-case selection introduces clear selection bias. By selecting only the two extreme cases and excluding the five intermediate athletes, the analytic sample was narrowed and the apparent phenotypic contrast was amplified; thus, these hypotheses cannot be generalized even to other finishers of the 217-MUM. Second, the performance outcomes can be confounded by body composition. The slower athlete was clinically obese (40.27% body fat) while the faster was eutrophic; consequently, distinguishing exercise-induced GM adaptation from obesity-driven microbial dysbiosis is not possible. Third, in-race nutrition and hydration were unmeasured. The race duration differed by over 70 h between the two athletes. Discrepancies in total energy, carbohydrate, and fiber intake, as well as the timing of the last meal prior to T1 sampling, represent major confounders. These unmeasured dietary and transit variables could drive the observed functional and metabolite shifts completely independently of the microbiome's true adaptive capacity. Finally, shotgun metagenomics predicts functional potential but cannot confirm actual metabolic flux or enzyme activity.

## 5. Conclusions

This study provides descriptive, hypothesis-generating observations from two highly contrasting cases under an extreme real race condition. We observed that the normal-BMI fast-finisher exhibited smaller observed differences among the displayed pathways and selected enzyme-coding genes between samples collected before and after race completion, compared to the obese slow-finisher. This contrast co-occurred with divergent SCFA trajectories in samples collected after the race. The findings cannot be generalized even to other finishers in the same event. Future studies with larger cohorts, predefined analytical groups, detailed control of dietary and fluid intake, serial fecal sampling, gastrointestinal symptom monitoring, multi-omics integration, and host-related physiological outcomes are needed to clarify whether microbial functional potential is mechanistically associated with responses to ultramarathon race.

## Data Availability

All relevant data are available at https://www.ncbi.nlm.nih.gov/bioproject/PRJNA1045616/ and https://www.ncbi.nlm.nih.gov/bioproject//PRJNA 999191/.
